# CCL19-sorted mature dendritic cells have enhanced lymph node migratory capacity and function

**DOI:** 10.1186/1476-9255-12-S1-P2

**Published:** 2015-04-16

**Authors:** Michelle L Le Brocq, Alasdair R Fraser, Gerard J Graham, John D M Campbell

**Affiliations:** 1 Chemokine Research Group, Institute of Infection, Immunology and Inflammation, University of Glasgow, Glasgow, G12 8TA, UK; 2 Scottish National Blood Transfusion Service, Cellular Therapeutics Research Group, Edinburgh, EH17 7QT, UK

## 

Cell therapy regimens are frequently compromised by low-efficiency cell homing to therapeutic niches. Improvements in this regard would enhance effectiveness of clinically-applicable cell therapy. The major regulators of tissue-specific cellular migration are chemokines and therefore selection of therapeutic cellular populations for appropriate chemokine receptor expression would enhance tissue-homing competence. We have recently shown that biotinylated ligands for chemokine receptors can be used as efficient cell sorting reagents[1]. These ligands have several advantages over antibodies, as they can be chemically synthesized to the appropriate grade for clinical use; produced and modified extremely quickly and are generally not species-specific. Furthermore, receptors are rapidly recycled post cell selection leaving cells free to respond to chemokine gradients, and thereby migrate to the required therapeutic site. We have applied this technology to the improvement of dendritic cell (DC) and T cell products for potential use as cellular therapeutics. CCL19, a high-affinity ligand for CCR7, is of particular interest in this field, as CCR7 expression by DC is a marker of maturity and lymph node homing ability. Also, CCR7^+^ central memory T cells are the most desired cell population for generation or engineering of tumour-antigen-specific therapeutic responses. Here we report the generation of biotinylated, chemically synthesised, modified human CCL19 molecules for optimised cell sorting. Our results demonstrate that these ligands allow the efficient enrichment of mature, antigen-loaded human or murine CCR7^+^ DC using a rapid and established magnetic bead sorting strategy. CCR7^+^ murine bone marrow-derived DC (BM-DC) isolated using this GMP-compliant method display significantly improved lymph-node homing ability *in vivo* over standard (unsorted) BM-DC preparations. Using *in vitro* models, such as murine OT-I, we also demonstrate the ability of these sorted DC to efficiently generate both effector and memory antigen-specific T cell responses. In conclusion, we show that biotinylated ligands for chemokine receptors constitute a novel, GMP-compliant cell sorting strategy that could enhance the efficacy of existing clinical cell therapy regimens.
